# Rheumatic Heart Disease among Patients with Valvular Heart Disease Admitted to the In-patient Department of a Tertiary Care Centre: A Descriptive Cross-sectional Study

**DOI:** 10.31729/jnma.7457

**Published:** 2022-05-31

**Authors:** Kriti Basnet, Shreeyash Raj Bhattarai, Sangam Shah, Amir Joshi, Sanjit Kumar Sah, Roshan Gami, Rajaram Khanal

**Affiliations:** 1 Maharajgunj Medical Campus, Institute of Medicine, Tribhuvan University, Maharajgunj, Kathmandu, Nepal; 2 Tribhuvan University Teaching Hospital, Maharajgunj, Kathmandu, Nepal; 3 Department of Cardiology, Manmohan Cardiothoracic Vascular and Transplant Centre, Maharajgunj, Kathmandu, Nepal

**Keywords:** *epidemiology*, *prevalence*, *rheumatic heart disease*

## Abstract

**Introduction:**

Valvular heart disease continues to cause significant morbidity and mortality around the world, with rheumatic heart disease accounting for the bulk of cases in developing nations. The aim of this study is to find out the prevalence of rheumatic heart disease among patients with valvular heart disease admitted to the in-patient department of a tertiary care centre.

**Methods:**

A descriptive cross-sectional study was conducted from December, 2018 to December, 2020 at a tertiary care centre after receiving ethical approval from the Institutional Review [Reference number: 395 (6-11) e^2^ 077/078]. Patients of age >18 years presenting with valvular manifestations of any disease diagnosed by transthoracic echocardiography were included and patients other than Nepalese nationals were excluded. Convenience sampling was done and a sample size of 327 was taken. Data were collected, entered and analyzed using the Statistical Package for the Social Sciences version 22.0. Point estimate at a 95% Confidence Interval was calculated along with frequency and percentages for binary data.

**Results:**

Among 327 patients, the prevalence of rheumatic heart disease was found to be 237 (72.47%) (67.63-77.31 at 95% Confidence Interval).

**Conclusions:**

The prevalence of rheumatic heart disease was similar to the other similar studies conducted in similar settings.

## INTRODUCTION

A cardiovascular condition affecting one or more of the four heart valves is known as valvular heart disease (mitral, tricuspid, aortic, and pulmonary valve). It could be inherited or acquired. Congenital heart illnesses have an unknown etiology, whereas acquired causes include rheumatic heart disease, degenerative heart disease, infective endocarditis, and a variety of other conditions.

In developed countries, degenerative valve illnesses are more common, whereas rheumatic heart disease is more common in developing countries.^1-3^ Because of the degenerative nature of the valves, the frequency of valvular heart disease rises significantly with age.^1^

Studies regarding the prevalence of rheumatic heart disease and higher studies are lacking in our country.

The aim of this study is to find out the prevalence of rheumatic heart disease among patients with valvular heart disease admitted to the in-patient department of a tertiary care centre.

## METHODS

A descriptive cross-sectional study was conducted in Manmohan Cardiothoracic Vascular and Transplant Centre (MCVTC) among patients with valvular heart disease. The patients with valvular heart disease admitted to the MCVTC from December, 2018 to December, 2020 were enrolled in our study. Data was collected after receiving ethical approval from the Institutional Review Committee of the Institute of Medicine (IOM) [Reference number: 395 (6-11) e^2^ 077/078].

We recruited all the patients with valvular heart disease at the MCVTC during the study duration. Only those patients of age ≥18 years presenting with valvular manifestations of any disease diagnosed by transthoracic echocardiography were included. Only the first examination recording was selected when the patients underwent multiple echocardiography sessions during this period. We excluded all patients other than Nepalese nationals and those under 18 years of age. The patients who showed valvular lesions after their admission to the hospital in the second echocardiography were also excluded from our study. The sample size was calculated using the following formula:

n = (Z^2^ × p × q) / e^2^

  = (1.96^2^ × 0.50 × 0.50) / 0.06^2^

  = 267

Where,

n = minimum required sample size
Z = 1.96 at 95% Confidence Interval (CI)
p = prevalence taken as 50% for maximum sample size calculation
q = 1-p
e = margin of error, 6%

The minimum required sample size was 267. A 10% was added to address the non-response rate, after which the required sample size was 294. However, a sample size of 327 was taken for the study. A structured data sheet was used to collect the data from the hospital records. We took written informed consent from the patients that were admitted to MCVTC. Data regarding the age, sex, diagnosis of the disease, name of the valve involved (mitral, aortic, tricuspid, and pulmonary), type of valvular lesion (stenotic and regurgitant), and severity of valve lesion (mild, moderate, and severe) were noted.

The etiology and classification of valvular heart disease were carried out according to the 2014 American Heart Association (AHA)/ American College of Cardiology (ACC) Guidelines.^4^ The diagnosis of rheumatic heart disease was made based on the revised Jones criteria.^5^ The severity of Mitral Stenosis (MS) was measured by planimetry and that of Aortic Stenosis (AS) by pressure gradient and continuity equation. Detection and gradation of valvular regurgitation were made on the basis of pressure half time for Aortic Regurgitation (AR) and Proximal Isovelocity Surface Area (PISA) for Mitral Regurgitation (MR). Data were collected from the hospital record for the past 2 years of MCVTC.

Data were entered and analyzed using the Statistical Package for the Social Sciences version 22.0. Point estimate at a 95% Confidence Interval was calculated along with frequency and percentages for binary data.

## RESULTS

Among 327 patients studied, the prevalence of Rheumatic Heart Disease (RHD) was found to be 237 (72.47%) (67.63-77.31 at 95% Confidence Interval). Isolated MS and MR were the most common valvular presentation of rheumatic heart disease seen in 18 (7.59%), and 11 (4.64%) respectively. There were no reported cases of isolated Tricuspid Stenosis (TS) and Pulmonary Regurgitation (PR). Isolated Pulmonary Stenosis (PS) was reported only in 1 (0.42%). MS in combination with Tricuspid Regurgitation (TR) was the most common mixed valvular presentation seen in 30 (12.65%) (Table 1).

**Table 1:** Valvular lesions seen among patients with rheumatic heart disease (n= 237).

Isolated valvular lesions	n (%)
MS	18 (7.59)
MR	11 (4.64)
AS	4 (1.69)
AR	4 (1.69)
TS	-
TR	5 (2.10)
PS	1 (0.42)
PR	-
**Mixed valvular lesions**	
MR+TR	30 (12.65)
MS+MR	27 (11.39)
MS+TR	20 (8.43)
MR+AR	14 (5.90)
TR+AR	8 (3.37)
MS+AR	10 (4.21)
AS+AR	4 (1.69)
MR+AS	2 (0.84)
MS+AS	3 (1.26)
MS+MR+TR	30 (12.65)
MR+AR+TR	21 (8.86)
MS+AR+TR	25 (10.54)

The mean age group of the population was 40.08±14.80 years, with 147 (62.02%) people falling in the age group of 18-44 years. There were 73 (30.80%) males and 164 (69.19%) females (Table 2).

**Table 2:** Demographic profile of the patients (n = 237).

Sex	Age group (years)	n (%)
	18-44	41 (17.29)
	45-54	18 (7.60)
**Males**	55-64	11 (4.64)
	65-74	3 (1.26)
	75 and above	-
	18-44	106 (44.70)
	45-54	30 (12.65)
**Females**	55-64	22 (9.28)
	65-74	3 (1.26)
	75 and above	3 (1.26)

Mitral valve involvement was the commonest with MR seen in a total of 66 (27.84%) out of which 36 (15.18%) were severe MR and MS seen in a total of 80 (33.75%) out of which 40 (50%) were severe MS (Table 3).

**Table 3:** Valvular lesions among the patients with RHD (n = 237).

Valvular lesion	Age group (years)	n (%)	Severe lesions n (%)
**MR**	18-44	48 (20.25)	25 (10.54)
	45-54	9 (3.79)	5 (2.10)
	55-64	9 (3.79)	5 (2.10)
	65-74	2 (0.84)	1 (0.42)
	75+	1 (0.42)	-
	Total	66 (27.84)	36 (15.18)
**TR**	18-44	25 (10.54)	13 (5.48)
	45-54	10 (4.21)	6 (2.53)
	55-64	9 (3.79)	7 (2.95)
	65-74	2 (0.84)	2 (0.84)
	75+	2 (0.84)	-
	Total	48 (20.25)	28 (11.81)
**AR**	18-44	20 (8.43)	11 (4.64)
	45-54	5 (2.10)	4 (1.68)
	55-64	4 (1.68)	-
	65-74	3 (1.26)	-
	75+	1 (0.42)	-
	Total	33 (13.92)	15 (6.32)
**AS**	18-44	5 (2.10)	2 (0.84)
	45-54	3 (1.26)	2 (0.84)
	55-64	1 (0.42)	1 (0.42)
	65-74	1 (0.42)	1 (0.42)
	75+	-	-
	Total	10 (4.21)	6 (2.53)
**MS**	18-44	48 (20.25)	24 (10.12)
	45-54	16 (6.75)	8 (3.37)
	55-64	13 (5.48)	6 (2.53)
	65-74	2 (0.84)	1 (0.42)
	75+	1 (0.42)	1 (0.42)
	Total	80 (33.75)	40 (16.87)

## DISCUSSION

Rheumatic heart disease accounted for 72.47% of the patients with valvular heart disease in the study. Studies have shown RHD as a very common cause of valvular heart disease in Southeast Asian countries including India^6^ and Sri Lanka.^7^ However, in developed countries where the burden of non-communicable disease is more common, age-related degenerative valvular heart disease is a more common cause of valvular heart disease.

Our study showed the age group 18-44 years consists of the greatest number of patients with valvular heart lesions which then decreased with age. However, in developed countries, the trend has been found to be the opposite with the prevalence of valvular heart disease decreasing with the increase in age. This is due to the high prevalence of RHD in developing nations which affects the younger age group population.^6,8^ Similar findings were found in a study done in Nepal.^9,10^ Consistent patterns of valve disease distribution reveal that older age is an independent determinant of all types of valve disease that occur often in the elderly, supporting the assumption that the burden of valve illness rises with age.

Our study has found that regarding the prevalence of RHD, females are the predominant sex (69.19% of all females had RHD in comparison to 30.80% of males). The prevalence of females in RHD has also been found higher in other studies done in India.^6,8^ In the Indian subcontinent, RHD continues to affect millions of children and young people, with prevalence rates ranging from 4.54 to 6 per 1000^11^ with prevalence estimations as high as 51 per 1000 in certain studies,^12^ despite recent large series of school surveys showing a reduction in RHD prevalence (0.5-0.68 per 1000).^13,14^

Studies have shown that the high prevalence of clinically significant valvular heart disease is equally notable in the population for clinically diagnosed valve disease. Undiagnosed valve disorders are most likely to blame for the disparity between the prevalence of such diseases in the population and the percentage of patients diagnosed with valvular heart disease in the community (25% vs 18%).^15^ One-fifth of initially uncomplicated RHD cases aged 35 years advanced to death/non-fatal complication within 8 years, according to a study, and the complication risk was highest within the first 6 months after RHD diagnosis.^16^

Valve disorders found in the general population and diagnosed in the community are not just imaging anomalies; they are linked to pressure or volume overload and are independent predictors of eventual mortality in both contexts. As a result, these findings emphasise the significance of our findings as well as the need for more research and treatment efforts directed at valvular diseases. Valve disorders affect men and women equally in the population, but women are identified less frequently in the community, implying a diagnostic imbalance that could have major consequences for women with valve diseases. Age >14 years, metropolitan residence, and a prior Acute Rheumatic Fever (ARF) record were all found to be independent predictors of RHD progression, although sex and population group were not in an Australian cohort.^16^

During the 2-year follow-up of persons with RHD, the Global Rheumatic Heart Disease Registry (REMEDY) study in low- and middle-income countries in Africa and Asia reported death rates between 12.5% and 20.8%, heart failure rates between 6.1% and 9.0%, and surgery rates between 3.1% and 13.0%.^17^ In comparison to lower-income countries, upper-middle-income countries had the lowest rates of death and cardiovascular problems, but the greatest rates of surgical intervention. Given the over-representation of advanced RHD in REMEDY, the higher progression rates were expected, whereas the lower surgical intervention rates are likely because of limited access to surgical or percutaneous valvular treatments.^17^

Our findings contrast with those of another valvular heart disease echocardiographic study, the Euro Heart Survey,^18^ a multicentre study encompassing 5001 patients from 92 locations in 25 European nations. The most common lesions were AS and MR, both of which were caused by degenerative processes. This disparity reflects the disparity in the prevalence of valve disease between industrialized and developing countries. In the current investigation, valve lesions in RHD were found in the following order: mitral, aortic, tricuspid, and pulmonary. This illustrates the pathogenic involvement of the heart valves in acute rheumatic fever, with mitral valve involvement being the most common and pulmonary valve involvement being the least common.^19^

In the current investigation, mitral stenosis was the commonest isolated valvular lesion seen in 18 (7.59%) of patients with RHD. Similar reports were shown in studies done in Nepal where MS was the most common valvular heart lesion in RHD.^9,10^ Surgical pathology data have demonstrated rheumatic involvement in over 99 percent of excised stenotic valves,^20,21^ which is consistent with the findings of a vast necropsy series (100%).^22^ Congenital MS, which occurs in around 1% of cases, is uncommon in adults since the median age at death is only 2 months.^23^ Degenerative calcification of the mitral annulus is a common cause of MR, but it can also cause mitral valve stenosis in about 3% of patients.^24^

The second most prevalent isolated valve lesion was MR. It was similar to the two surgical series, where rheumatic involvement was the most common etiology seen in 31%^20^ and 38%^25^ respectively, followed by myxomatous or mitral valve prolapse (40% and 33% respectively). The peak incidence of rheumatic MR occurred two decades before the peak incidence of MS. This could be due to the fact that, following an acute incident of rheumatic fever, there is usually a considerable latent period before the stenotic mitral lesion manifests clinically.^26^ In the current investigation, MR was more prevalent than MS in the childhood age group (19 years), although MS was twice as common in people aged 20 years.

A shifting pattern of aortic stenosis pathology may be seen in three surgical series from 1965 to 1990, with the degenerative disease now being the most common etiology of AS.^27^ In the older Mayo Clinic surgical series,^28^ RHD was the most common cause of solitary AR, while in the more current series,^27^ aortic dilatation and/or degenerative valve alterations were responsible for half of the cases. In this study, 4 (1.69%) of the 237 patients had pure AS, 4 (1.69%) had pure AR, and the rest had both AS and AR in 4 (1.69%). This is akin to an 85-case natural history autopsy series which reported that 72% of bicuspid valves had developed stenosis.^29^

Multivalvular illness (anatomic or hemodynamic combinations of lesions) was fairly common in our study, occurring in more than a third of patients. The different incidence of mitral and aortic valve combinations in the adult (≥20 years) age groups was a notable finding in our study. In a study of the juvenile age group, MR+AR was more common than MS+AR, but the opposite was true in the adult age group, indicating the natural progression of valve lesions following an attack of severe rheumatic fever. A study of 100 patients with combined aortic and mitral valve replacement discovered that MR + AR was the most common combination of rheumatic valvular diseases.^30^ Only 10 patients were reported to have virtually pure AS and AR in a group of patients with mixed aortic and mitral valve disease.^31^

RHD has been shown to be the most common cause of TS, which was virtually always found in conjunction with MS. Despite the fact that 12% showed some pathological involvement of the tricuspid valve, stenosis of the valve was found in only 2% of cases in a necropsy series, and only when associated MS and AS were present.^32^ RHD was the most common cause of pure TR in a surgical pathology series of tricuspid valve disease, followed by Ebstein's abnormality (41% and 14%, respectively);^33^ the equivalent results in our study was 64.9% by RHD.

There were a few limitations in our research. A study based in a tertiary referral facility, for example, is more likely to include symptomatic lesions. Patients with more severe lesions are likely to be overrepresented in research done in tertiary care referral centres; hence there may be a bias toward the severity patterns of lesions. Despite the application of precise morphologic and clinical criteria for the diagnosis, an electrocardiography-based diagnosis has inherent limitations when compared to surgical or autopsy-based studies.

## CONCLUSIONS

In our study, the prevalence of rheumatic heart disease was similar to the other studies conducted in similar settings. RHD is the most common cause of valvular heart disease in Nepal. The diagnosis made on the basis of clinical evaluation may not be enough to detect the early valve lesions. Thus, early screening by echocardiography and prophylactic therapy can reduce the need for valvular repair and surgery for diseases like RHD in countries like Nepal.
